# Death Does Matter—Cancer Risk in Patients With End-Stage Renal Disease: A Nationwide Population-Based Study With Competing Risk Analyses

**DOI:** 10.1097/MD.0000000000002512

**Published:** 2016-01-22

**Authors:** Shih-Feng Weng, Yu-Hsien Chiu, Ren-Long Jan, Yi-Chen Chen, Chih-Chiang Chien, Jhi-Joung Wang, Chin-Chen Chu

**Affiliations:** From the Department of Healthcare Administration and Medical Informatics, Kaohsiung Medical University, Kaohsiung (S-FW, Y-HC); Department of Pediatrics, Chi Mei Medical Center, Liouying (R-LJ); Graduate Institute of Clinical Medicine, National Cheng Kung University (R-LJ); Department of Medical Research (Y-CC); Department of Nephrology, Chi-Mei Medical Center (C-CC); Department of Food Nutrition, Chung Hwa University of Medical Technology (C-CC); Department of Anesthesiology, Chi-Mei Medical Center (J-JW, C-CC); and Department of Recreation and Health-Care Management, Chia-Nan University of Pharmacy and Science, Tainan, Taiwan (C-CC).

## Abstract

Patients with end-stage renal disease (ESRD) have a high mortality rate. We hypothesized that not accounting for death as a competing risk overestimates the event rate caused by ESRD. Thus, we examined the cancer risk for patients with ESRD (ESRD^Pos^) after death as a competing risk event had been adjusted for. Patients with newly diagnosed ESRD (n = 64,299) between 1999 and 2007, together with age- and sex-matched controls without ESRD (ESRD^Neg^) (n = 128,592) were enrolled (1:2). In a Cox proportional hazards model that included death as a competing risk, ESRD^Pos^ patients in Taiwan had a lower overall incidence (subdistribution hazard ratio [sdHR] = 1.29) of cancer than did ESRD^Neg^ patients in a Cox model that did not include death as a competing risk (HR = 1.70). After competing mortality had been adjusted for, ESRD^Pos^ patients ≥70 (sdHR = 0.82) and ESRD^Pos^ patients on long-term dialysis (> 5 follow-up years, sdHR = 0.62), had a lower risk for developing cancer than did ESRD^Neg^ patients. This finding supported our hypothesis that standard survival analyses overestimate the event rate, especially when the mortality rate is high. It also showed that ESRD^Pos^ patients, when they grow older, were far less likely to develop cancer and far more likely to die because of underlying illnesses that might also affect the risk of death because of ESRD.

## Abstract 2

Supplemental Digital Content is available in the text

## INTRODUCTION

Because of better dialysis therapy, the lifespan of patients with end-stage renal disease (ESRD^Pos^) is longer. Malignancy, however, has emerged as a significant complication in these ESRD^Pos^ patients. The causes of this cancer have remained unspecified, but they are probably related to imbalances in the immune and DNA repair systems, long-term chronic infection, and a decrease in antioxidant capacity.^1,2^ These are also characteristics of patients on dialysis. Many large-scale studies on cancer in White patients on dialysis have been done.^3,4^ Other small-scale studies have also been done in Asian countries.^5–7^ In general, patients on dialysis often develop kidney and bladder cancers.^3–5,7,8^ The incidence of other cancers is different in various population groups. Whites develop thyroid cancer more often than do other races,^3^ gastrointestinal cancer occurred more often in Korea,^5^ and liver cancer occurred more often in Taiwan,^9^ which has the world's highest proportion of patients on dialysis.^10^

All of above-cited studies on estimating the cancer risk of patients on dialysis were done using the standardized incidence ratio (SIR) or a hazard ratio (HR) analysis. However, these statistical methods might yield biased results when death from ESRD also affects the risk for cancer. Using the SIR means that the follow-up data might not be accurate and is almost inevitably biased, because some but not all patients on dialysis had undergone kidney transplantation but were not excluded, and patients censored because of death were not excluded when using general population rates to estimate the expected number of deaths.^11^ Some studies^12^ have used survival analyses to calculate an HR; however, patients who were not diagnosed with cancer and who were censored because of death were excluded from the risk set. Censoring in a standard survival analysis is treated as “noninformative,” which means that some participants drop out of the study for reasons unrelated to the study.^11^ When the disease has a high mortality rate, however, death should be considered a competing risk event in the analyses.

Competing risks commonly occur in medical research. The Kaplan–Meier method for estimating cumulative incidence, the log-rank test for comparing survival curves, and the standard Cox proportional hazards model for assessing covariates are used to analyze time-to-event data. These methods are appropriate when there is only a single type of event. However, when an individual undergoes another event that we are not considered, the likelihood of undergoing the event of interest mostly changes, which causes a competing risk problem.^13^ For example, in a study of head and neck cancer treated with chemoradiotherapy,^14^ death and second primary tumors are competing risk factors. Second, primary tumors are not analyzed after a patient has died. The presence of competing risks complicates the analysis of time-to-event, and standard survival analyses are not always appropriate and should be carefully interpreted.^15^ Standard survival analyses might overestimate the event rate, especially when the rate of the competing risk is high.^16,17^ Fine and Gray^18^, therefore, proposed the proportional subdistribution hazards model to overcome the problem of competing risk. In their method, covariate effects on cumulative failure probability were measured directly owing to one risk accompanies other risks. Similar to any other regression analysis, modeling cumulative incidence functions for competing risks could be used to identify latent prognostic factors for a specific event in the presence of competing risks.

Research on analyzing the risk of cancer between ESRD patients and the general population while considering death as a competing risk event was not extensive. We examined whether the cancer risk for patients with ESRD is similar after death as a competing risk event has been adjusted for.

## METHODS

### Database

Our data source was the Taiwan National Health Insurance Research Database (http://nhird.nhri.org.tw/en/Background.html), which provides detailed information about healthcare services (outpatient visits, hospitalizations, procedures, and prescriptions) rendered, claimed, and subsidized by the NHI for each patient. For each outpatient visit and hospitalization, the data contain dates and up to 3 to 5 diagnoses coded under the International Classification of Diseases, Ninth Revision, Clinical Modification (ICD-9-CM), plus information about prescribed drugs and doses, treatments, and procedures. The Institutional Review Board of Chi Mei Medical Center approved this study and waived the requirement of informed consent because all personal information in the NHIRD is de-identified, which protects patients and their families from having their privacy rights violated.

### Study Sample

The inclusion criteria for ESRD^Pos^ patients were as follows: (i) first dialysis treatment began after 31 December 1998, (ii) maintained on regular dialysis for >90 days, and (iii) reconfirmed by being given a catastrophic illness certificate (CIC) with the ICD-9-CM code 585 between 1 January 1999 and 31 December 2007. ESRD^Pos^ patients who developed cancer were defined as those who also received the CIC with the ICD-9-CM code 140–208 after their first dialysis. ESRD^Pos^ patients who were diagnosed with cancer before their first dialysis or who had undergone a kidney transplantation were excluded. Each ESRD^Pos^ patient was followed-up to determine the incidence of cancer until the end of 2008 or censored because of death. Because it is customary for many Taiwanese to “die at home,” “in-hospital death” coded at discharge usually underestimates true hospital mortality. Therefore, except when “in-hospital death” was coded, we also presumed an in-hospital death for patients who withdrew from the NHI program within 30 days of hospital discharge because emigration, another reason for withdrawal, is highly unlikely shortly after a severe disease. In such a circumstance, the discharge date was designated as the date of death.

### Control Group

For each ESRD^Pos^ patient, we randomly selected 2 ESRD^Neg^ controls from the Longitudinal Health Insurance Database 2000 (LHID2000), which contains all original claims data and registration files (from 1996 to 2008) for 1,000,000 enrollees randomly selected from the system in 2000. There are no differences in age, sex, or average insured payroll-related premiums between the LHID2000 sample group and all NHI enrollees. We matched the controls by sex, age, and index date. The index date for the ESRD^Pos^ patients was the date of their first dialysis, and the index date for the controls was generated by matching the date with the ESRD^Pos^ patients’ index date. ESRD^Neg^ controls diagnosed with cancer before the index date were excluded. To determine the incidence of cancer, each patient was followed-up until the end of 2008 or censored because of death.

### Cancer Risk in Competing Risk Analysis

Each patient was tracked from the index date to define whether he or she acquired the cancers (see Table, Supplemental Content, which illustrates corresponding ICD-9 CM-codes for cancer and major comorbidities). Moreover, the NHI program claims pathological validation of a cancer, validated cases must be reported in the Catastrophic Illness Patient Database. Therefore, we linked those identified patients to the registry database for a reconfirmation of the diagnosed cancers. Demographic characteristics and comorbidities were also recorded. Because the risk of cancer can be affected by other critical factors, major baseline comorbidities were collected, including hypertension (HTN), diabetes mellitus (DM), chronic coronary artery disease (CAD), and stroke (see Table, Supplemental Content, which illustrates corresponding ICD-9 CM-codes for cancer and major comorbidities). These data were enrolled only if the comorbidity occurred either in an inpatient setting or in at least 3 ambulatory care claims coded 12 months before the index medical care date. Patients who developed cancer during the first 3 months were excluded. To reduce the selection bias between the ESRD^Pos^ and ESRD^Neg^ groups, the status of comorbidities was adjusted in our regression model.

Death usually resulted from an underlying illness that might also affect the risk of ESRD, and its occurrence led to informative censoring in estimating the incidence of cancer. Therefore, death that occurred before cancer was diagnosed was considered a competing risk event in analysis. We used a modified Cox proportional hazards model that included the presence of a competing risk event so that we could examine the independent association between ESRD and cancer.

### Statistical Analysis

Pearson χ^2^ tests were used to compare the demographics and comorbidities of the ESRD^Pos^ and ESRD^Neg^ groups. After we had confirmed the assumption of proportional hazards, we estimated the risk of getting cancer using Cox proportional hazards models while considering death a competing risk event and adjusting for age, sex, baseline comorbidities, and geographic distribution. We used a modified Kaplan–Meier method and the Fine and Gray^18^ method to compare cumulative incidence. The SAS (SAS Institute, Cary, NC) PSHREG macro^19^ was used to calculate the cumulative incidence and the subdistribution hazards ratio (sdHR). The SAS macro PSHREG applied SAS's standard Cox regression procedure, PROC PHREG, using weights and counting-process style of specifying survival times. It can be used to fit a proportional sdHR model for survival data with competing risks. SAS 9.4 was used for all analyses. Significance was set at *P* < 0.05 (2-sided).

## RESULTS

### Demographic Data

Based on NHIRD claims data, between 1999 and 2007, there were 64,299 ESRD^Pos^ patients who met the eligibility criteria for this study. For the control group, 128,598 ESRD^Neg^ age- and sex-matched patients were randomly selected. The demographic characteristics for the 2 groups were not significantly different (Table 1). There were significant differences in the baseline comorbidities of DM, HTN, CAD, and stroke between the groups (*P* < 0.0001). The mean age of the ESRD^Pos^ patients when they were diagnosed with cancer was 63.57 ± 12.13 years, and of the ESRD^Neg^ patients was 69.42 ± 10.52 years (*P* *<* 0.0001). The mean interval between the index date and the occurrence cancer was 3.10 ± 2.17 years in the ESRD^Pos^ patients and 3.59 ± 2.28 years in ESRD^Neg^ patients (*P* *<* 0.0001) (Table 1).

**TABLE 1:**
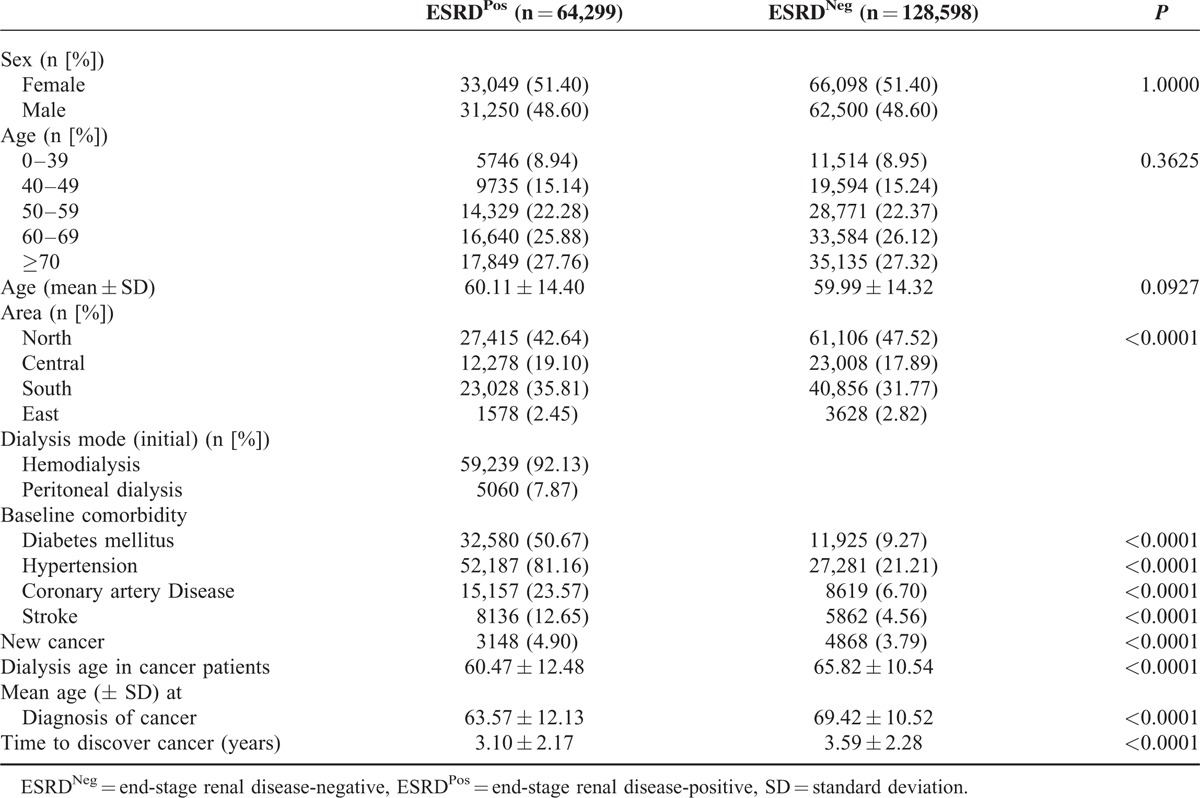
Demographic Characteristics and Comorbid Medical Disorders for ESRD^Pos^ and ESRD^Neg^ Patients in Taiwan

### Correlation of Patient Characteristics With the Overall Risk of Developing Cancer

ESRD^Pos^ patients in Taiwan had a significantly (*P* < 0.001) higher overall rate of cancer than did ESRD^Neg^ patients in both Cox proportional hazards models, the one that did and the one that did not count death as a competing risk (Table 2). In this stratification analysis, younger ESRD^Pos^ patients on dialysis had a significantly higher risk of developing cancer (0–39 years old [_sd_HR = 4.24, 95% CI = 3.22–5.59; *P* < 0.0001; HR = 4.55, 95% CI = 3.45–6.00; *P* < 0.0001], 40–49 years old [sdHR = 2.84, 95% CI = 2.48–3.25; *P* < 0.0001; HR = 3.21, 95% CI = 2.80–3.68; *P* < 0.0001], 50–59 years old [sdHR = 1.90, 95% CI = 1.73–2.10; *P* < 0.0001; HR = 2.32, 95% CI = 2.10–2.55; *P* < 0.0001], and 60–69 years old [sdHR = 1.08, 95% CI = 1.00–1.17; *P* < 0.533; HR = 1.43; *P* < 0.0001]) compared with the ESRD^Neg^ group. However, although the risk of developing cancer in the ≥70 group (HR = 1.15, 95% CI = 1.06–1.26; *P* = 0.0008) was significantly higher in ESRD^Pos^ patients than in ESRD^Neg^ patients, the risk of developing cancer adjusted for competing mortality was significantly lower (sdHR = 0.82, 95% CI = 0.75–0.89; *P* < 0.0001). The risk of developing cancer was also significantly different based on sex (male sdHR = 1.13, 95% CI = 1.06–1.20; *P* = 0.0001; HR = 1.50, 95% CI = 1.41–1.60; *P* < 0.0001], female [sdHR = 1.50, 95% CI = 1.40–1.60; *P* < 0.0001; HR = 1.93, 95% CI = 1.81–2.06; *P* < 0.0001]). A stratified analysis of the follow-up period indicated a higher risk of developing cancer in the first follow-up year after dialysis (sdHR = 1.85, 95% CI = 1.65–2.07; *P* < 0.0001). This risk declined in the following 1 to 3, 3 to 5, and >5 follow-up years groups. In the group with a follow-up period >5 years, the risk of developing cancer even decreased (sdHR = 0.62, 95% CI = 0.56–0.69) in the ESRD^Pos^ patients after competing mortality had been adjusted for, whereas the risk of developing cancer without a competing mortality adjustment in the ESRD^Pos^ group was 1.54 times (HR = 1.54, 95% CI = 1.39–1.69) higher than that in the ESRD^Neg^ group.

**TABLE 2:**
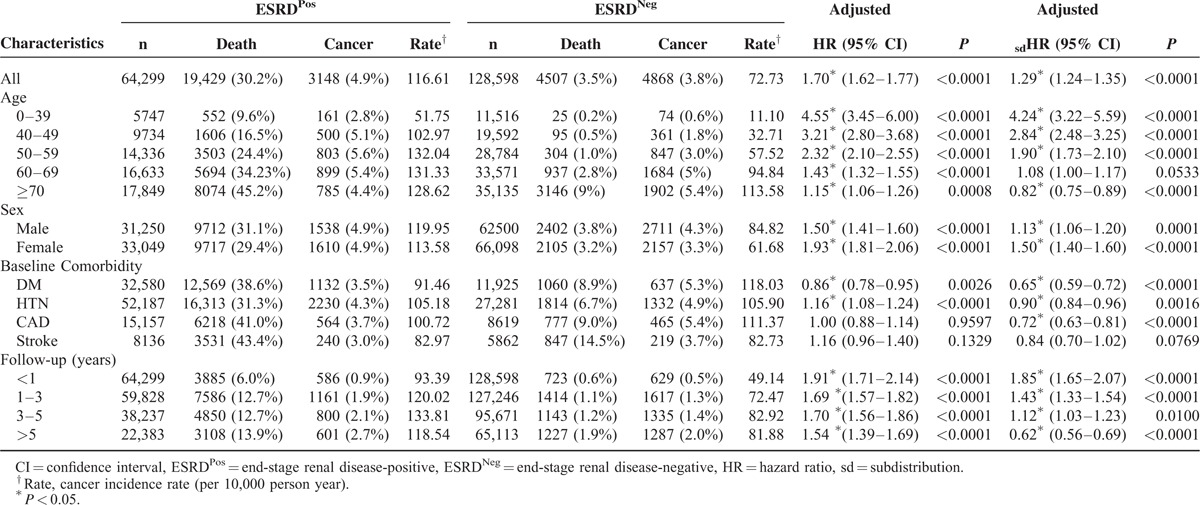
Stratification Analysis: Multivariable Cox Proportional Hazards Model Analysis for Risk of Developing Cancer Without and With Adjusting Competing Mortality

### Cumulative Incidences of Cancer for ESRD^Pos^ and ESRD^Neg^ Patients

**FIGURE 1:**
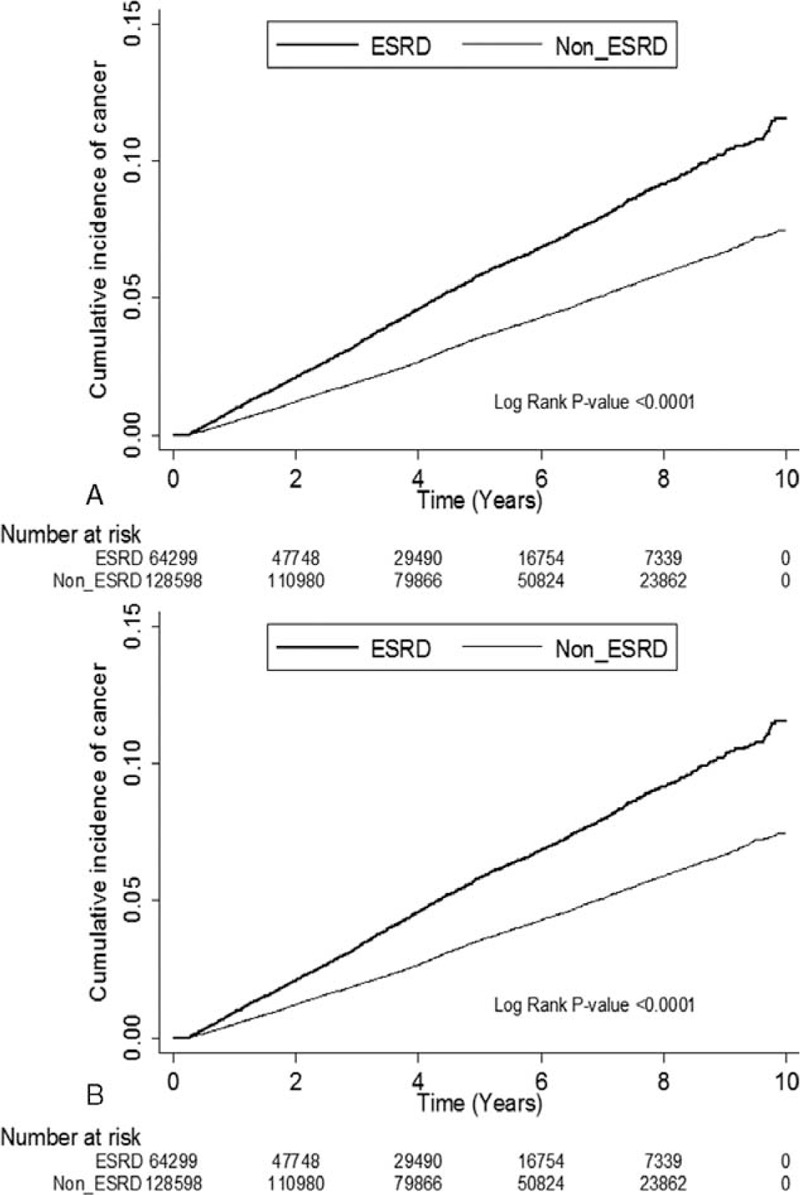
(A) Cumulative incidence of cancer estimated using the Kaplan–Meier method without accounting for competing risk events. (B) Cumulative incidence of cancer estimated using the Kaplan–Meier method and accounting for competing risk events.

There were significant differences in the cumulative incidences of cancer in the log-rank tests with and without the competing mortality adjustment between the ESRD^Pos^ and ESRD^Neg^ patients (Figure 1A and B; both *P* < 0.001). ESRD^Pos^ patients had a significantly higher risk of developing cancer than did ESRD^Neg^ patients. The 1-, 5-, and 10-year cumulative incidences of cancer without the competing mortality adjustment were 0.94% versus 0.49%, 6.02% versus 3.62, and 12.28% versus 7.76%, respectively, in the ESRD^Pos^ patients compared with the ESRD^Neg^ patients. In contrast, the 1-, 5-, and 10-year cumulative incidences of cancer with the competing mortality adjustment were 0.75% versus 0.57%, 4.76% versus 3.65%, and 9.23% versus 7.12%, respectively, in the ESRD^Pos^ patients compared with the ESRD^Neg^ patients.

### Cancer Distribution and Risk for Developing Cancer after Competing Mortality Had Been Adjusted for

Bladder cancer had the highest incidence (644/3148, 20.00%) in ESRD^Pos^ patients, followed by liver cancer (488/3148, 15.50%), and then kidney cancer (454/3148, 14.42%) (Table 3). A multivariable Cox proportional hazards model analyzed with and without considering death as a competing risk and adjusted for sex, age, baseline comorbidities, and geographic distribution yielded an adjusted sdHR of 1.29 (95% CI = 1.24–1.35) and an HR of 1.70 (95% CI = 1.62–1.77) between ESRD^Pos^ and ESRD^Neg^ patients. Moreover, bladder (sdHR = 6.33, 95% CI = 5.41–7.41) and kidney (sdHR = 8.45, 95% CI = 6.84–10.44) cancer had the top 2 adjusted HRs between ESRD^Pos^ and ESRD^Neg^ patients, followed by liver cancer (sdHR = 1.45, 95% CI = 1.29–1.63). Although most cancers showed a higher incidence in ESRD^Pos^ patients, the incidences of prostate cancer (sdHR = 0.38, 0.28–0.51), lung cancer (sdHR = 0.55, 95% CI = 0.47–0.65), and cancer of the digestive organs and peritoneum (sdHR = 0.75, 95% CI = 0.64–0.87) were lower.

**TABLE 3:**
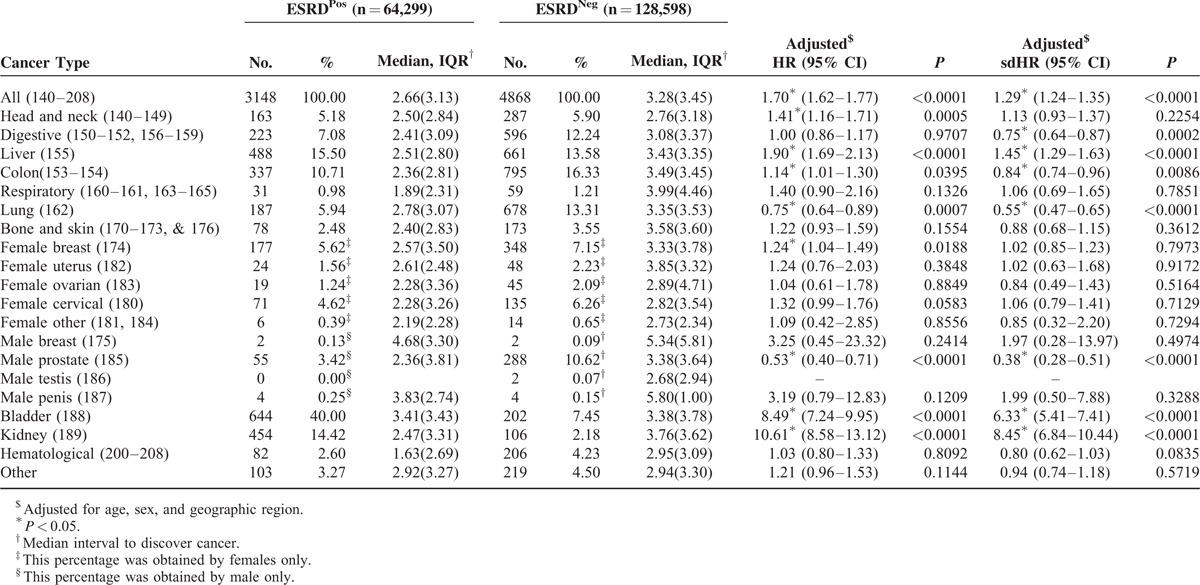
The Competing Risk of Specific Cancers for ESRD^Pos^ and ESRD^Neg^ (Controls) Patients in the Follow-Up Period

The median period for diagnosing cancer in ESRD^Pos^ patients (median = 2.66 years, interquartile range [IQR] = 3.13 years) from the index date was significantly (*P* < 0.0001) shorter than that in ESRD^Neg^ patients (median = 3.28 years, IQR = 3.45 years). Diagnosing respiratory and hematological cancers required the shortest period (Table 3).

Because of treating age as a continuous variable such as age, we might miss some information, which might affect the results. However, in our study, when age was treated as a continuous variable, the hazard ratio of developing cancer between ESRD^Pos^ and ESRD^Neg^ patients remained the same as when age was treated as a categorical variable (sdHR = 1.29, 95% CI = 1.26–1.32) (Table 4).

**TABLE 4:**
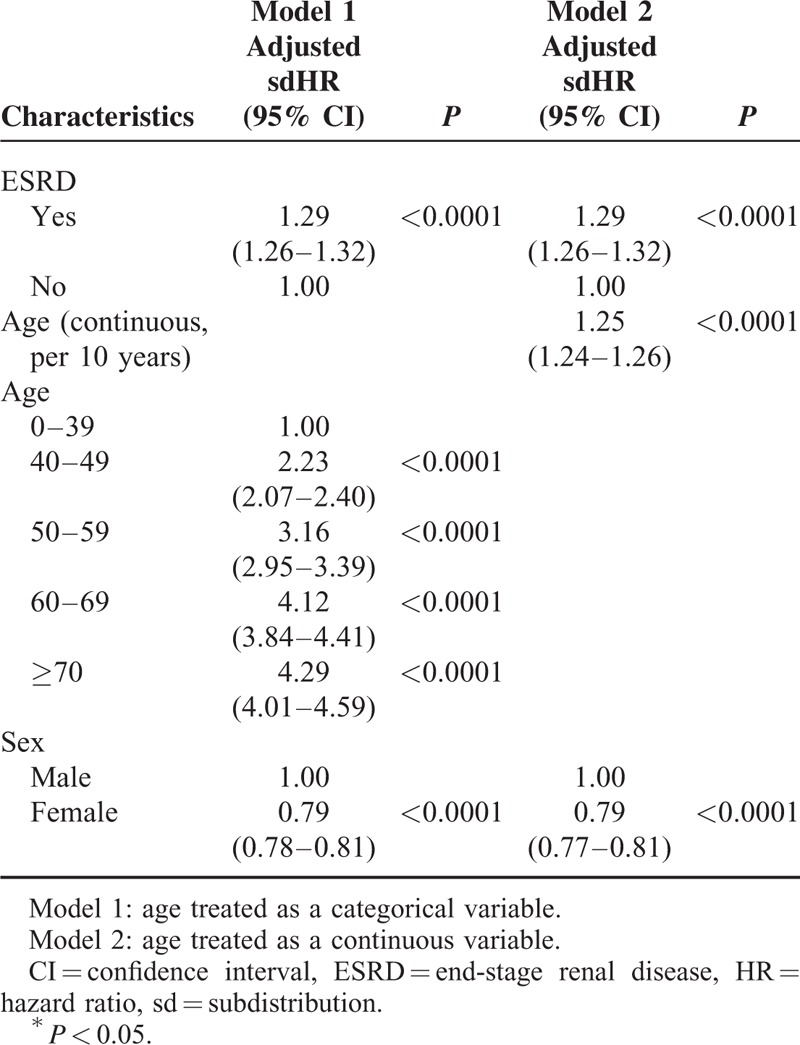
Multivariable Cox Proportional Hazards Model Analysis for Risk of Developing Cancer With Competing Risk Analysis in Different Model

## DISCUSSION

We found that the risk of cancer in ESRD^Pos^ patients in Taiwan was lower in a Cox proportional hazards model that included death as a competing risk (sdHR = 1.29) than in one which did not (HR = 1.70). One possible reason for this is that standard survival analyses overestimate the event rate, especially when the rate of competing risks is high.^16,17^

### The Effect of Old Age on the Risk of Cancer

In our study, although the incidence rate (IR) of cancer in ESRD^Neg^ patients increased when their age increased, the highest incidence rate of cancer in ESRD^Pos^ was in patients 50 to 59 years old (IR = 132.04/10,000 person years). The reason the IR did not increase as age increased for ESRD^Pos^ patients was the high death rate of the patients in this group. Moreover, the association between cancer and ESRD decreased with age, even when patients were ≥70 years old (sdHR = 0.82; 95% CI = 0.75–0.89, *P* < 0.0001). Standard survival analyses, however, still showed that ESRD^Pos^ patients had a higher risk than did ESRD^Neg^ patients (HR = 1.15, 95% CI = 1.06–1.26, *P* = 0.0008). The “death as a competing risk” analysis showed that ESRD^Pos^ patients were far more likely to die than to develop cancer when they grew older (death rate for patients ≥70 years old: ESRD^Pos^ vs ESRD^Neg^ = 45.2% vs 9%), which was similar to the finding of Lin et al^9^: elderly patients on dialysis had a lower SIR of cancer than did their healthy age-matched counterparts. Liang et al^12^ also reported a lower HR in the ESRD control group of patients ≥70. These findings indicated that cancer prevention should focus on younger ESRD^Pos^ patients. The risk of cancer was higher in all ESRD^Pos^ than in all ESRD^Neg^ patients. Moreover, the risk in female ESRD^Pos^ patients was higher than in male ESRD^Pos^ patients, which is consistent with many studies.^3,9^

### The Effect of Longer Dialysis and Follow-up on the Risk of Cancer

The length of dialysis might also affect the risk of developing cancer. Maisonneuve et al^3^ reported that the SIR of ESRD^Pos^ patients on dialysis was 2.3 (95% CI = 2.0–2.6) for the first year, and, in Australia and New Zealand, was 1.0 (95% CI = 0.4–2.6) for >10 years and 1.2 (95% CI = 1.1–1.2) for the first year, and, in Europe, 0.9 (95% CI = 0.8–1.1). The longer the duration of dialysis, the lower the incidence of new cancer was. Lin et al^9^ also indicated that the longer the dialysis duration was, the lower the incidence rate of developing new cancer was (SIR = 8.3 [95% CI = 7.6–9.0] for the first year and SIR = 0.3 [95% CI = 0.2–0.3] for >10 years). However, this might be attributable to the increased detection of cancer at the time of the initial dialysis treatment (ascertainment bias).^3^ In contrast, for American ESRD^Pos^ patients, the longer their dialysis duration, the higher their incidence of new cancer was (SIR = 1.2 [95% CI = 1.1–1.2] for the first year and SIR = 1.4 [95% CI = 1.2–1.5] for >10 years).^3^ An important conflict between our findings and those of Maisonneuve et al^3^ is that, in our study, the longer the dialysis treatment, the lower the incidence rate was, and that the rate even declined after 5 years of dialysis. These findings suggested that efforts to prevent the development of cancer should begin with no delay after the initial dialysis, because ESRD^Pos^ patients are far more likely to die than to develop cancer when their dialysis time was longer (death rate after >5 years from first dialysis: ESRD^Pos^ vs ESRD^Neg^ = 13.9% vs 1.9%).

### Cancer Type After ESRD

We also found that ESRD^Pos^ patients on dialysis often developed kidney and bladder cancers, which is consistent with the findings of other studies.^3–5,7,8^ The incidence of other cancers might be different in different population groups, however. For example, Whites developed thyroid cancer more often than people in other ethnic groups,^3^ Koreans developed gastrointestinal cancer more often,^5^ and Japanese developed gynecological cancer more often.^6^ We found that urinary bladder, kidney, and liver cancer had the highest incidences, which was consistent with the findings of the other 2 studies,^9,12^ but with a lower sdHR after the competing mortality adjustment. Many large-scale studies have documented the high incidence rate of hepatoma in ESRD^Pos^ patients, especially in Taiwan, an endemic area of hepatitis B virus (HBV) and hepatitis C virus (HCV).^20,21^ However, because ESRD caused by HBV and HCV infection is a response to the development of hepatoma,^22,23^ ESRD^Pos^ patients with HBV and HCV should be matched and compared to confirm the risk of liver cancer. One study^24^ has reported that there was no significant difference in risk between ESRD^Pos^ patients and the general population after matching HBV and HCV without considering competing mortality.

ESRD^Pos^ patients had a lower incidence of lung and prostate cancer than did the general population, even after adjusting for competing mortality, a finding similar to those of Liang et al^12^ and Lin et al.^9^ Maisonneuve et al^3^ reported that the risk of developing prostate cancer was lower for ESRD^Pos^ patients in Europe and the United States but higher for those in Australia and New Zealand. ESRD^Pos^ patients also had a higher risk for lung cancer in Australia, New Zealand, and the United States, but a lower risk in Europe. Most studies^9,12,25^ say that there is no significant difference in colon cancer between ESRD^Pos^ patients and the general population, but Maisonneuve et al^3^ reported a significantly higher risk between ESRD^Pos^ patients and the general population in the United States. In our study, without adjusting for competing mortality, the HR was 1.14, but after adjusting for competing mortality, the sdHR was 0.84. Nevertheless, colon cancer in ESRD^Pos^ patients in Taiwan was the fourth most frequent type (10.71%); thus, colon cancer prevention plans are needed. Lee et al^5^ reported that dialysis-associated hematologic cancer developed more rapidly than did other cancers. In our study, hematological (median = 1.63, IQR = 2.69 years) and respiratory (median = 1.89, IQR = 1.56 years) cancer developed more rapidly than did other cancers. Although bladder and kidney cancer were the 2 most frequently developed cancers in ESRD^Pos^ patients, the median time intervals for discovering them were 3.41 (IQR = 3.43) years and 2.47 (IQR = 3.31) years, respectively.

### Strengths and Limitations

This large population-based dataset allowed us to estimate the risk for ESRD^Pos^ patients on dialysis to develop cancer. The large sample size increased the statistical power with a minimal selection bias for matching case and control groups. In addition, previous studies often used the SIR to estimate the risk, but the SIR might be inaccurate because, unlike our analysis, it does not include death as a competing event.

This study had some limitations. First, the NHIRD does not provide data about patients’ socioeconomic status, daily habits (eg, smoking and drinking), height, weight, ethnicity (eg, Han Chinese, Hakka, etc) or the results of patients’ medical (eg, blood pressure) and pathology (eg, blood cultures) reports. In addition, because the cause of death data is not linked to the NHRID, there is no way to determine whether a patient died from cancer or other diseases. A large percentage of the ESRD^Pos^ patients on dialysis died from cardiovascular diseases; however, we used their principal diagnosis code of the last hospitalization to estimate their cause of death.

## CONCLUSION

Because we included death as a competing event in our analysis, we found a positive association between the development of some cancers and ESRD. However, the cancer risk calculated with a competing mortality adjustment was lower than the risk without the adjustment. After competing mortality had been adjusted for, patients who were ≥70 years old and on long-term dialysis (>5 follow-up years), had a lower risk for developing cancer than did other ESRD^Pos^ and ESRD^Neg^ patients. This indicated that patients with ESRD and on dialysis were far more likely to die of other causes rather than develop cancer. Thus, the type of healthcare provided these 2 categories of patients is more important than their risk of developing cancer when they grow older.

## Supplementary Material

Supplemental Digital Content
