# Supplementary material for: Death Does Matter—Cancer Risk in Patients With End-Stage Renal Disease: A Nationwide Population-Based Study With Competing Risk Analyses
Source: Medicine (Baltimore). 2016 Jan 22;95(3):e2512. doi: 10.1097/MD.0000000000002512 (PMC4998265; doi:10.1097/MD.0000000000002512)
Supplement: Supplemental Digital Content [file medi-95-e2512-s001.doc]

Supplemental Content. Table that illustrates the corresponding ICD-9 CM codes for cancer and major comorbidities.

| Diagnosis | ICD-9-CM code |
| --- | --- |
| Cancer |  |
| Head and neck | 140-149 |
| Respiratory and intrathoracic organs | 160-161, 163-165 |
| Lung | 162 |
| Digestive organs and peritoneum | 150-152, 156-159 |
| Colon | 153-154 |
| Liver | 155 |
| Bone and skin | 170-173, 176 |
| Female breast | 174 |
| Male breast | 175 |
| Female cervical | 180 |
| Female uterus | 182 |
| Female ovarian | 183 |
| Female other | 181, 184 |
| Prostate | 185 |
| Testis | 186 |
| Penis | 187 |
| Bladder | 188 |
| Kidney | 189 |
| Hematological | 200-208 |
| Others | 166-169, 176-178, 190-192, 197-199 |
| Comorbidities |  |
| Hypertension (HTN) | 401-405 |
| Diabetes mellitus (DM) | 250 |
| Chronic coronary artery disease (CAD) | 410-414 |
| Stroke | 430-438 |

ICD-9 CM = International Classification of Diseases, Ninth Revision, Clinical Modification.
